# Patient blood management and surgery: Impact of new surgical techniques on transfusion needs

**DOI:** 10.1111/vox.70179

**Published:** 2026-02-09

**Authors:** Francois Ansart, France Pirenne

**Affiliations:** ^1^ Department of Digestive, Pancreatic and Endocrine Surgery AP‐HP Pitié‐Salpêtrière Hospital Paris France; ^2^ Etablissement Français du Sang, INSERM U955 University Paris Est Créteil Créteil France

**Keywords:** PBM, surgery, transfusion

## Abstract

**Background and Objectives:**

Patient blood management (PBM) is a multidisciplinary approach aimed at reducing the use of blood products. It considers the patient's own blood as a valuable resource to preserve and seeks to avoid the routine use of transfusions to treat anaemia. PBM is primarily based on interventions implemented by anaesthesiologists, including preoperative anaemia correction, targeted administration of coagulation factors and tranexamic acid and strict adherence to transfusion protocols. However, the role of surgical approaches in this context deserves attention. Indeed, surgical innovations over the past two decades have significantly contributed to reducing transfusion requirements. This review will focus on this aspect of PBM.

**Materials and Methods:**

For this review, we performed a comprehensive search of the PubMed database related to PBM in surgery and also consulted other relevant databases.

**Results:**

In the preoperative period, advances in diagnostic techniques, surgical indications and surgical access allow for two blood‐sparing options: active surveillance or alternatives to surgery. In the intraoperative period, the development of minimally invasive approaches, the use of innovative haemostatic techniques, per surgery autologous transfusion (cell salvage) and the prevention of hypothermia contribute to the reduction of blood loss and, consequently, the need for transfusion. In the postoperative period, proactive patient management through careful monitoring and the application of enhanced recovery after surgery principles plays a major role in decreasing overall postoperative morbidity.

**Conclusion:**

These innovations have significantly reduced the need for transfusion in surgical practice. They enhance patient safety by minimizing bleeding and transfusion‐related risks.

**Highlights**

- Patient blood management (PBM) is an approach to reducing the use of blood products by preserving the patient's own blood.
- Surgical innovations—such as minimally invasive techniques—advanced haemostasis, cell salvage, hypothermia prevention and enhanced recovery after surgery principles have markedly decreased perioperative blood loss.
- Surgical innovations improve patient safety by lowering transfusion‐related risks and overall morbidity.

## INTRODUCTION

Patient blood management (PBM) is a multidisciplinary approach designed to reduce the need for blood transfusion in patients. This strategy considers the patient's own blood as a vital resource to be preserved and aims to avoid systematic transfusion to correct anaemia. The concept was initially developed in Australia and the United Kingdom and has been officially recommended by the World Health Organization since 2010 [1]. Its adoption has expanded in response to evidence that anaemia, whether acute, as in postoperative cases, or chronic, is an independent risk factor for perioperative morbidity and mortality. While transfusion remains a mainstay in the management of anaemia, allogeneic blood transfusion is not without risks. Beyond the well‐documented complications such as alloimmunization, allergic reactions, transfusion‐related acute lung injury (TRALI) and transfusion‐associated circulatory overload (TACO), additional concerns have emerged under the concept of transfusion‐related immunomodulation (TRIM). TRIM has been associated with increased rates of postoperative infections and higher recurrence rates of some cancers, such as colorectal cancer following surgery [2, 3]. PBM is based on three pillars: optimizing the patient's blood reserves, minimizing blood loss and optimizing the patient's tolerance to anaemia. Optimization of the patient's blood reserves involves correcting preoperative anaemia and its underlying causes, including nutritional deficiencies. Improvement of anaemia tolerance is achieved through the application of strict transfusion threshold guidelines [4] while ensuring optimal oxygen delivery and consumption at the tissue level [1]. Reduction of blood loss is ensured through perioperative medical–surgical cooperation. From a medical standpoint, perioperative bleeding control and haemostasis monitoring [5], including the administration of platelets, coagulation factors, tranexamic acid and fibrinogen, have led to significant reductions in intraoperative blood loss and transfusion needs [1]. In parallel, over the last two decades, surgical innovation has also played a major role in decreasing transfusion requirements. This review will focus specifically on the contributions of surgical innovations to PBM.

## MATERIALS AND METHODS

To conduct this review, we performed a comprehensive search of the PubMed database without time restrictions, focusing on English‐language articles related to PBM in surgical settings. We selected the most relevant original studies and systematic reviews to support our discussion. Additionally, we consulted other relevant databases, particularly those reporting transfusion rates of blood products.

## REVIEW OF PBM IN SURGERY

### Evolution in transfusion requirements in surgery

**FIGURE 1:**
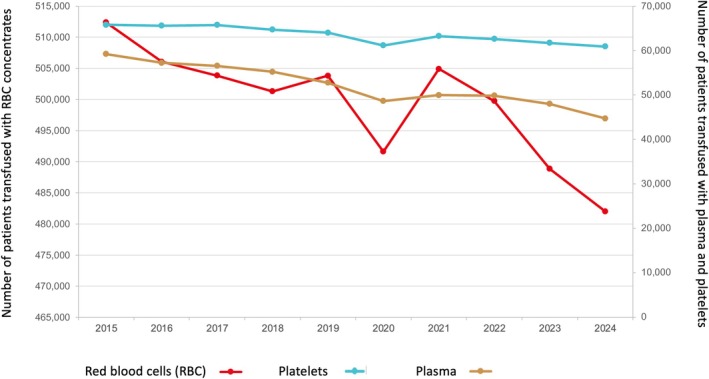
Evolution of the number of transfused patients in France by product and by year (data from the French Blood Establishment).

In France, approximately one‐third of all transfusions are administered in a surgical context, while oncology and haematology account for nearly half of all transfused patients [6]. Over the past decade, the number of red blood cell (RBC) units transfused in France has gradually declined (Figure 1). This trend is likely multifactorial and disease‐dependent. In non‐surgical specialties, particularly oncology and haematology, the development of new treatments has led to a reduction in the duration and severity of chemotherapy‐induced aplasia, thereby reducing transfusion needs [7, 8, 9]. According to the National Blood Collection and Utilization Survey in the United States, the number of RBC units transfused has markedly declined, from 14,465,000 units in 2001 to 10,328,000 units in 2023, reflecting a significant reduction in transfusion practices over time [10, 11, 12, 13, 14, 15, 16, 17].

Surgical practice has also contributed to the decrease in RBC transfusion rates. Over the past 20 years (2002–2022), transfusion rates in orthopaedic surgery have declined from 24%–25% to 0.4%–1.2% and in cardiac surgery from 60.1% to 26.7%. These improvements are largely attributable to technological innovations in surgery developed over the last two decades (Table 1).

**TABLE 1:** Impact of new surgical techniques on transfusion needs.

Surgery	Preoperative	Intraoperative	Postoperative	References
General	Active surveillance and reduction of surgical indications Alternative to surgery	Monitoring of bleeding and haemostasis Procoagulant medication Highly effective haemostatic techniques Haemostatic patch Cell salvage Hypothermia prevention	Active and anticipatory surveillance ERAS Reduction of surgical drainage	[18, 19] [20, 21, 22, 23, 24, 25] [5] [1] [38, 39] [40, 41] [42, 43, 44, 45] [46] [47] [48, 49] [50, 51, 52]
Digestive and pelvic		Minimally invasive: Laparoscopy or robotic surgery		[34, 35, 36, 37]
Orthopaedic and trauma		Minimally invasive: Mini‐incisions or arthroscopy Tourniquet release		[29, 30, 31] [31]
Urological		Minimally invasive: Robotic surgery		[32, 33]

Abbreviation: ERAS, enhanced recovery after surgery.

### Reducing surgical bleeding

#### Preoperative phase

In the preoperative phase, advances in diagnostic techniques, surgical indications and approaches offer two major opportunities for preservation of the patient's own blood: active surveillance and non‐surgical alternatives.

##### Active surveillance and reduced surgical indications

Advancements in medical imaging over the past three decades, especially its use for diagnosis and monitoring, have refined and reduced surgical indications. As demonstrated by Rao et al., improved use of computed tomography imaging has led to a >10% reduction in unnecessary appendectomies [18]. Similarly, management strategies for intraductal papillary mucinous neoplasms of the pancreas have evolved, favouring active surveillance under certain conditions over major surgery, which previously carried significant morbidity and mortality [19].

##### Non‐surgical alternatives

The development of interventional radiology has also reduced the need for conventional surgery. A meta‐analysis by Ahmad et al. involving 8698 patients compared transcatheter aortic valve implantation (TAVI) with surgical valve replacement. In low‐risk patients, TAVI was associated with a significant reduction in 1‐year morbidity and mortality [20]. In coronary revascularization, advancements in coronary angioplasty have significantly decreased the number of surgical bypass procedures [21]. In vascular surgery, endovascular techniques such as stent angioplasty offer effective arterial revascularization without open surgery [22]. In gastrointestinal surgery, endoscopy has become increasingly prevalent in diagnosing and treating conditions that previously required surgery [23, 24, 25].

#### Intraoperative phase

During surgery, innovations in access routes, advanced haemostatic technologies, intraoperative autologous transfusion (cell salvage) and hypothermia prevention have significantly reduced bleeding and transfusion needs.

##### Minimally invasive techniques

**FIGURE 2:**
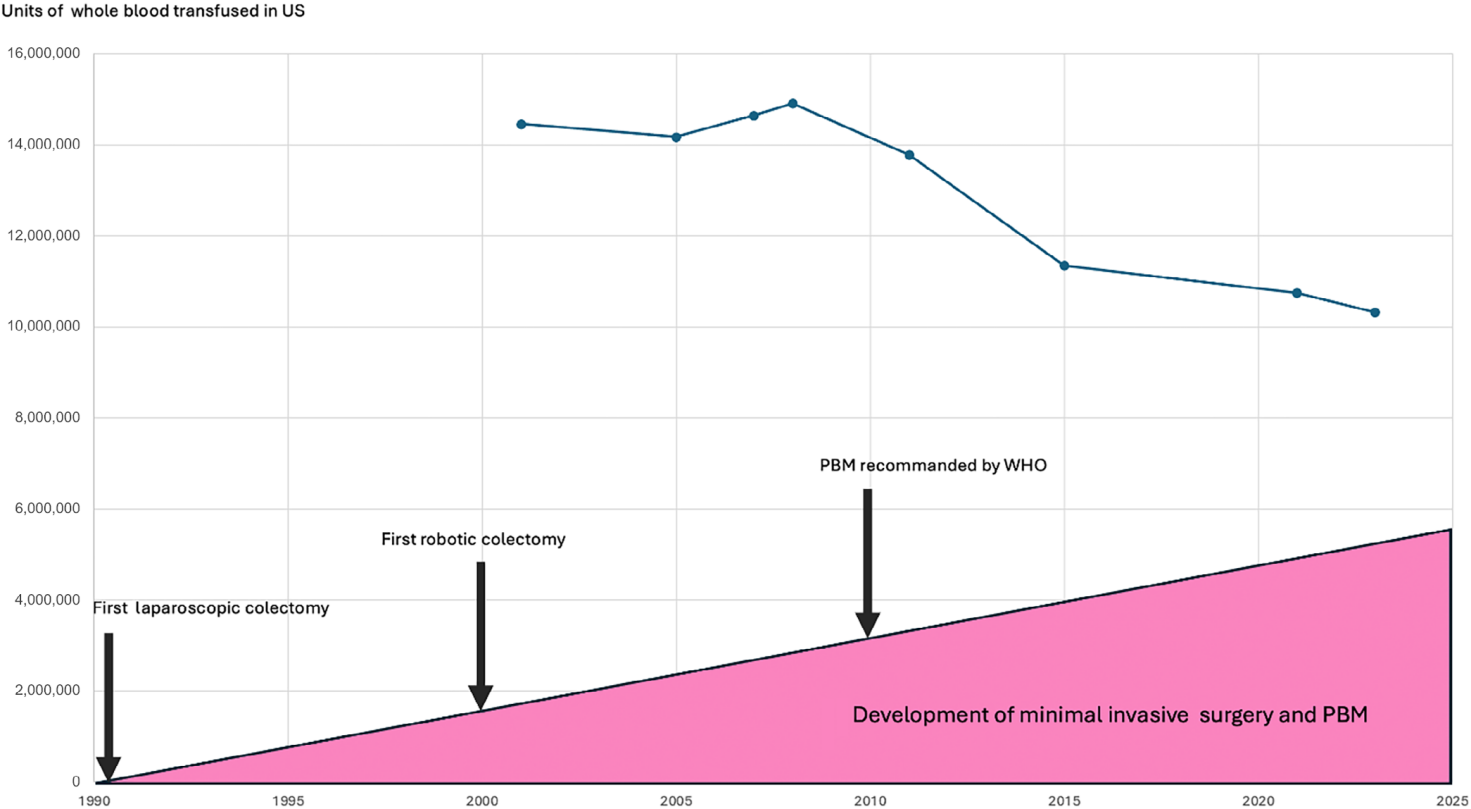
Impact of the development of minimally invasive surgery and the implementation of patient blood management (PBM) on transfusion needs in the United States. With the first laparoscopic colectomy performed in 1991 by Jacobs et al., the first robotic colectomy performed on 6 March 2001 by Weber et al. and PBM officially recommended by the World Health Organization in 2010.

Minimal invasive surgery, including laparoscopy and robotics, has contributed significantly to lowering perioperative morbidity and mortality, both in emergency [26] and elective settings [27, 28]. In orthopaedics, the use of mini‐incisions and arthroscopy reflects this evolution. A literature review on mini‐incisions for hip procedures demonstrated the advantages over standard incisions in terms of intraoperative blood loss, shorter operative time and reduced hospital stays [29]. Arthroscopy is now the standard approach for many orthopaedic conditions, particularly in the shoulder and knee [30, 31], including meniscal tears and septic arthritis. Conversely, the use of tourniquets in orthopaedics to reduce bleeding is now debated or abandoned, as it does not consistently reduce blood loss and may impair functional recovery while increasing the risk of deep vein thrombosis [31]. In urology, robotic surgery has demonstrated excellent results in reducing blood loss during prostate and bladder surgeries [32, 33]. Basiri et al. have shown that robot‐assisted radical prostatectomy reduces blood loss, transfusion needs and length of hospital stay [32]. In abdominal surgery, following the first laparoscopic colectomy performed in 1991 by Jacobs et al. and subsequently the first robotic colectomy performed in 2001 by Weber et al., minimally invasive approaches have progressively replaced many open procedures [34, 35]. Multiple studies have confirmed their superiority in terms of morbidity, pain and recovery in colorectal, hepatic and endocrine surgeries [36, 37]. These advances are summarized in Figure 2.

##### Advanced haemostatic techniques

Since the 1990s, new haemostatic tools such as advanced bipolar electrosurgery and ultrasonic scalpels have enabled highly effective tissue coagulation and dissection, reducing both bleeding and operative time [38, 39]. Haemostatic patches, whether mechanical or biologically active (e.g., collagen‐ or fibrin‐based), have also demonstrated benefit in controlling bleeding [40, 41].

##### Intraoperative autologous transfusion (cell salvage)

Close collaboration between anaesthesiologists and surgeons is crucial. Intraoperative cell salvage (autologous transfusion) exemplifies this synergy. In obstetrics, systematic use of cell salvage in haemorrhagic scenarios has been shown to significantly reduce the need for allogeneic transfusions [42]. A minimum of 500 mL must be collected for clinical benefit, limiting its use to high‐risk procedures such as liver transplantation, cardiovascular, obstetric or trauma surgery [43]. Studies show greater blood‐saving benefits in trauma than in cardiac surgery [44, 45].

##### Prevention of perioperative hypothermia

Maintaining normothermia during surgery is also essential. Hypothermia has been identified as a risk factor for impaired coagulation [46]. A 1°C drop in body temperature increases bleeding risk by 16% and transfusion need by 22% [46]. Hypothermia induces coagulopathy via reduced coagulation factor efficiency, impaired platelet function, splenic or hepatic platelet sequestration and loss of coagulation factors through bleeding. Guidelines from the French Society of Anaesthesia and Intensive Care (SFAR) recommend warming blankets, warm irrigation fluids and warmed intravenous fluids to maintain core temperature above 36.5°C.

#### Postoperative phase

##### Anticipatory monitoring

Optimized postoperative surgical management, including regular clinical assessment and routine lab work, plays a key role. Active monitoring and prompt decision making regarding reintervention based on clinical and imaging findings are essential [47].

##### Enhanced recovery after surgery

The enhanced recovery after surgery (ERAS) approach aims to improve postoperative recovery and reduce perioperative morbidity [48, 49]. Recommendations from French health authorities include multimodal analgesia, early feeding and mobilization and limited use of surgical drains. Drainage is increasingly questioned outside of specific indications, such as cardiovascular or certain abdominal surgeries. Some authors suggest that drains neither improve early detection of bleeding or anastomotic leaks nor enhance prognosis; instead, they may prolong hospital stay and increase postoperative morbidity [50, 51, 52].

In conclusion, advances in diagnostic imaging, development of innovative surgical techniques such as laparoscopy and robotic surgery, introduction of highly effective haemostatic tools, selective use of intraoperative autologous transfusion (cell salvage), prevention of hypothermia and adherence to ERAS protocols have collectively reduced transfusion needs in surgery over the past two decades (Figure 2). These innovations not only improve patient safety by minimizing bleeding and transfusion‐related complications but also contribute to public health by lowering the overall cost of surgical care.

## CONFLICT OF INTEREST STATEMENT

The authors declare no conflicts of interest.

## Data Availability

The data that support the findings of this study are available in the public domain. These data were derived from the PubMed database (https://pubmed.ncbi.nlm.nih.gov/). All references used in this review are listed in the References section of the manuscript.
